# The hemerythrin-like diiron protein from *Mycobacterium kansasii* is a nitric oxide peroxidase

**DOI:** 10.1016/j.jbc.2022.101696

**Published:** 2022-02-10

**Authors:** Zhongxin Ma, Ashley A. Holland, Ilana Szlamkowicz, Vasileios Anagnostopoulos, Maria Luiza Caldas Nogueira, Jonathan D. Caranto, Victor L. Davidson

**Affiliations:** 1 Burnett School of Biomedical Sciences, College of Medicine, University of Central Florida, Orlando, Florida, USA; 2 Department of Chemistry, University of Central Florida, Orlando, Florida, USA

**Keywords:** catalase, mycobacteria, nonheme iron, nitric oxide oxidase, nitric oxide dioxygenase, nitrosylation, tuberculosis, EPR, electron paramagnetic resonance, HLP, hemerythrin-like protein, Mka, *Mycobacterium kansasii*, NO, nitric oxide

## Abstract

The hemerythrin-like protein from *Mycobacterium kansasii* (Mka HLP) is a member of a distinct class of oxo-bridged diiron proteins that are found only in mycobacterial species that cause respiratory disorders in humans. Because it had been shown to exhibit weak catalase activity and a change in absorbance on exposure to nitric oxide (NO), the reactivity of Mka HLP toward NO was examined under a variety of conditions. Under anaerobic conditions, we found that NO was converted to nitrite (NO_2_^−^) *via* an intermediate, which absorbed light at 520 nm. Under aerobic conditions NO was converted to nitrate (NO_3_^−^). In each of these two cases, the maximum amount of nitrite or nitrate formed was at best stoichiometric with the concentration of Mka HLP. When incubated with NO and H_2_O_2_, we observed NO peroxidase activity yielding nitrite and water as reaction products. Steady-state kinetic analysis of NO consumption during this reaction yielded a *K*_m_ for NO of 0.44 μM and a *k*_cat_/*K*_m_ of 2.3 × 10^5^ M^−1^s^−1^. This high affinity for NO is consistent with a physiological role for Mka HLP in deterring nitrosative stress. This is the first example of a peroxidase that uses an oxo-bridged diiron center and a rare example of a peroxidase utilizing NO as an electron donor and cosubstrate. This activity provides a mechanism by which the infectious *Mycobacterium* may combat against the cocktail of NO and superoxide (O_2_^•−^) generated by macrophages to defend against bacteria, as well as to produce NO_2_^−^ to adapt to hypoxic conditions.

Certain mycobacteria possess oxo-bridged diiron proteins with active site features similar to those of hemerythrin (1). These are a distinct class of hemerythrin-like proteins (HLPs) that are found in mycobacterial species that cause respiratory disorders in humans. Unlike true hemerythrins (2), these HLPs do not function as oxygen carriers or oxygen-storage proteins. The first of these mycobacterial HLPs to be characterized was the Rv2633c protein from *Mycobacterium tuberculosis* (3). That protein was shown to exhibit catalase activity. Subsequently, the crystal structure was determined of the orthologous protein from *Mycobacterium kansasii* (4). This HLP from *M. kansasii* (Mka HLP) exhibited weak catalase activity and an additional reactivity toward nitric oxide (NO), as judged by an NO-dependent change in its absorbance spectrum (4). Understanding the precise activity of this protein is important, because the gene for the orthologous protein in *M. tuberculosis* is rapidly upregulated after phagocytosis of the bacteria by macrophages during infection (5, 6). As NO is generated in the macrophage to kill the infectious *Mycobacterium*, elucidation of the precise reactivity of the Mka HLP toward NO is of particular interest and could support future design of antimycobacterial drugs.

Hemerythrins and HLPs share a common structural feature of a four α-helix bundle that contains the oxo-bridged diiron site. However, their overall structures vary, as do the identity of the ligands that coordinate the two irons. The structure of the Mka HLP (4) shows it to be a monomer comprised of a similar four helix bundle with an additional fifth helix (Fig. 1*A*). The oxo-bridged diiron site in the mycobacterial HLPs is coordinated by the side chains of six amino acids; four histidines, two glutamic acids, and a tyrosine (1, 4) (Fig. 1*B*). The use of a tyrosine ligand and the pattern of amino acid ligation are conserved among the mycobacterial HLPs. These features are not seen in hemerythrins or HLPs from any other sources (1, 7). A bridging solvent oxygen completes the coordination. The coordination environment leaves one iron coordinatively saturated and the other with one open coordination site, which could accommodate NO or H_2_O_2_ binding.

**Figure 1:**
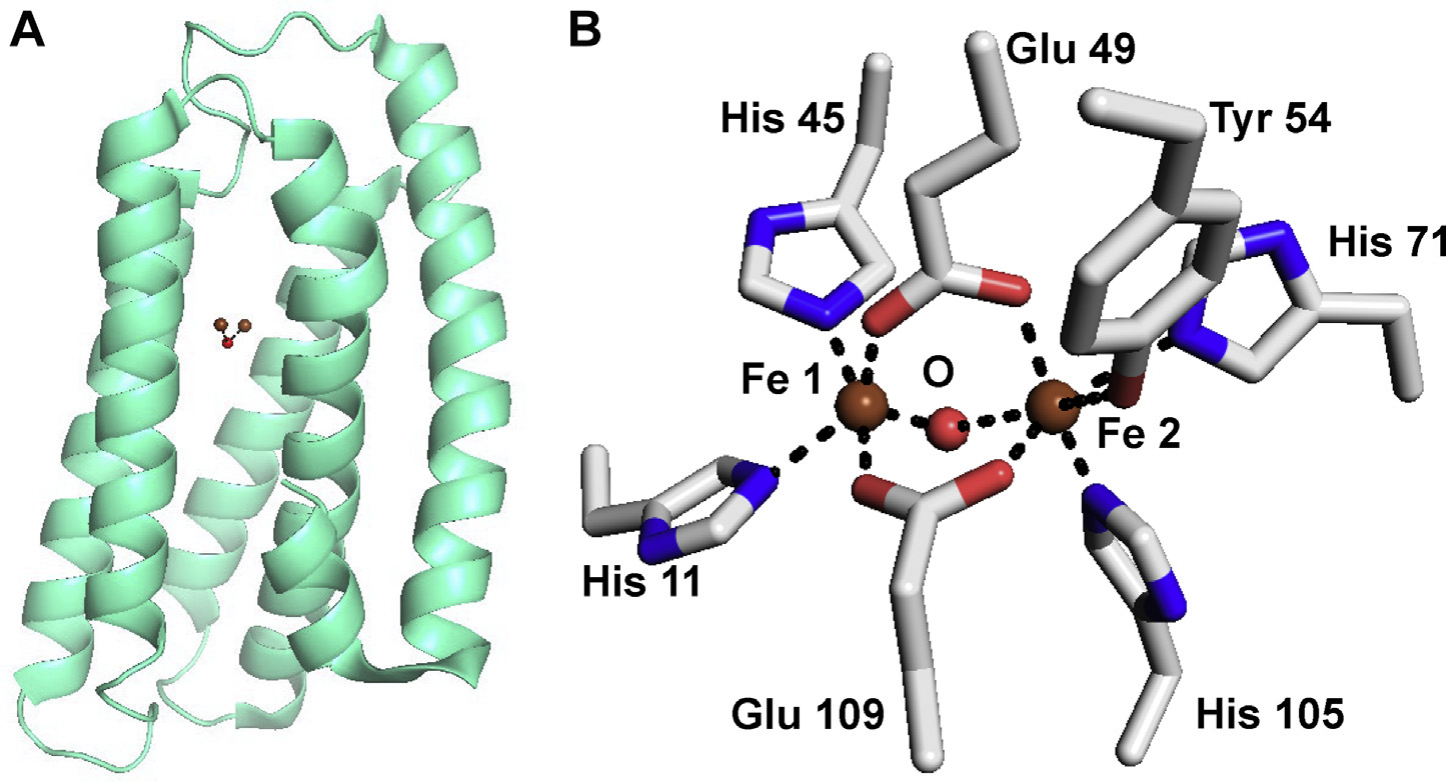
**Structure of the Mka HLP.***A*, cartoon of the overall structure with the irons and bridging oxygen indicated. *B*, the oxo-bridged diiron site and amino acid ligands. HLP, hemerythrin-like protein; Mka, *Mycobacterium kansasii*.

The previously observed NO-dependent change in the absorbance spectrum of the Mka HLP (4) is noteworthy as NO is used as a defense against mycobacterial infections. A lethal cocktail of NO and superoxide (O_2_^•−^) is generated by macrophages in response to the mycobacterial infections (8). Disproportionation of O_2_^•−^ results in a large reservoir of H_2_O_2_, which can participate in Fenton chemistry that causes lethal DNA damage. In addition, NO reacts with O_2_^•−^ to form peroxynitrite (ONOO^−^), which subsequently decomposes to form radical species that also mediate lethal DNA damage. Some pathogenic bacteria express NO detoxification enzymes to survive this host response (8, 9). These enzymes most commonly exhibit NO reductase or NO dioxygenase activities (10, 11). Thus, it is likely that reaction of NO with the Mka HLP also protects the infectious *Mycobacterium* from this host defense mechanism. An NO oxidase could convert NO to nitrite (NO_2_^−^). An NO dioxygenase could convert NO to nitrate (NO_3_^−^). While rare, NO peroxidase activity could convert NO to nitrite (12, 13). Enzymes catalyzing these reactions typically use heme iron (10, 14). The NO reactivity of the Mka HLP is atypical for a nonheme diiron site and thus of mechanistic as well as physiological relevance.

This study examined the reactivity of the Mka HLP under a variety of reaction conditions to determine the exact nature of the reactivity toward NO and its possible physiological relevance. Under anaerobic conditions, some NO was converted to NO_2_^−^, and under aerobic conditions, some NO was converted to NO_3_^−^. However, in each case, the Mka HLP was unable to catalyze multiple turnovers. Significantly, the Mka HLP does function as an effective high-affinity NO peroxidase with catalytic formation of NO_2_^−^ and conversion of H_2_O_2_ to water. This is the first example of a peroxidase that uses an oxo-bridged diiron center, rather than a heme for catalysis, and is a rare example of a peroxidase that utilizes NO as an electron donor. Consistent with the role of NO as an electron donor for the peroxidase reaction, evidence is also presented that NO can react with one of the ferric irons and transfer an electron to generate Fe(II) *via* a process known as reductive nitrosylation (15). To our knowledge, this reactivity is unprecedented for a nonheme iron protein. In fact, binding of NO to the Fe(III) center of a nonheme iron protein, which is a prerequisite for reductive nitrosylation, has not been reported (16). The observed NO peroxidase activity of the Mka HLP, which is likely common to mycobacterial HLPs, would provide an activity that is an ideal defense mechanism for protection from NO and H_2_O_2_, which are produced in the macrophage during infection to combat the invading *Mycobacterium* (9). Furthermore, the NO_2_^−^ produced in the peroxidase reaction is a signaling molecule in mycobacteria that allows adaptation to the hypoxic conditions that are experienced by the *Mycobacterium* within the macrophage (17).

## Results

### Oxidation state of the Mka HLP

**Figure 2:**
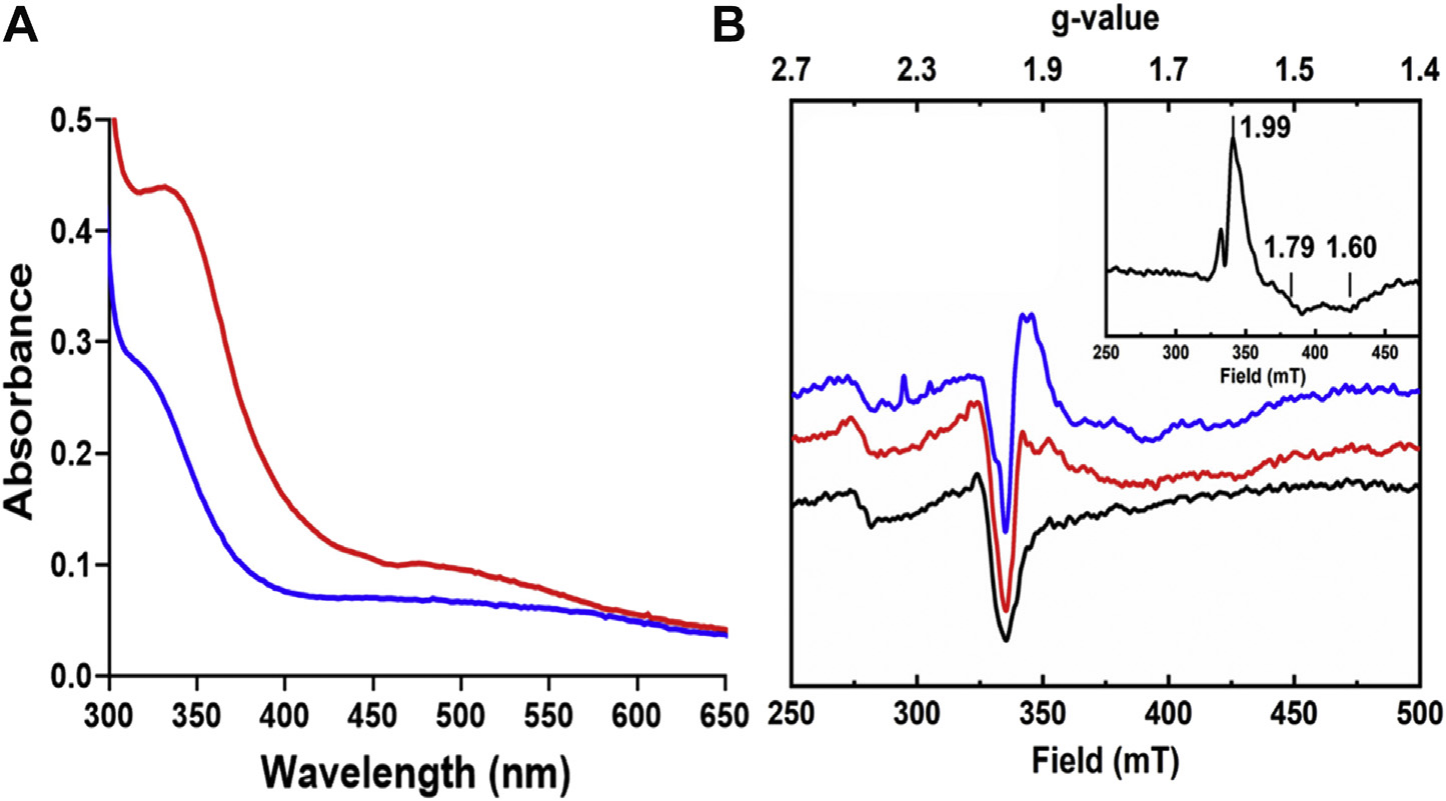
**Spectroscopic features of the as-isolated and dithionite-reduced Mka HLP.***A*, absorbance spectra of the Mka HLP. The spectra of the protein were recorded as-isolated (*red*) and after addition of dithionite (*blue*). *B*, EPR spectra of the Mka HLP. The spectra of the protein were recorded as-isolated (*red*) and after addition of dithionite (*blu*e). The background spectrum of the cavity is *black*. Spectra were collected at 1 mW and 17 K. The inset shows a spectrum of the dithionite-reduced sample after subtraction of the cavity signal with the g values indicated. These spectra were collected at 5 mW and 17 K. HLP, hemerythrin-like protein; Mka, *Mycobacterium kansasii*.

The absorbance spectrum of the as-isolated Mka HLP exhibits a 350-nm feature (Fig. 2*A*) that is characteristic of an oxo-bridged nonheme diferric site (18). Absorbance changes on addition of dithionite to reduce the protein were difficult to interpret as absorbance in this region remained, even after lengthy incubations with excess dithionite. This suggested that the Mka HLP could not be fully reduced to a diferrous site. Electron paramagnetic resonance (EPR) spectroscopy established the oxidation state. The as-isolated Mka HLP lacks any EPR signal. This result is consistent with an EPR silent species such as an antiferromagnetically coupled diferric state or a diferrous center (Fig. 2*B*). These two possibilities are differentiated in dithionite-reduced samples of the Mka HLP. Addition of one equivalent of dithionite results in a sharp rising feature with g-values of 1.99, 1.79, and 1.60. That these values are less that 2.0 is consistent with the presence of a mixed-valence diiron center (Fe^II^–Fe^III^). It follows that the as-isolated Mka HLP is in the diferric oxidation state. Surprisingly, even after treatment with additional reducing equivalents, only the 1-electron reduction of the diiron site to the mixed-valence state is observed. This suggests that the protein favors the mixed-valence oxidation state over the diferrous oxidation state. This finding distinguishes the Mka HLP from true hemerythrins that cycle between diferric and diferrous states (2, 19).

### Anaerobic reaction of the Mka HLP with NO to form NO_2_^−^

**Figure 3:**
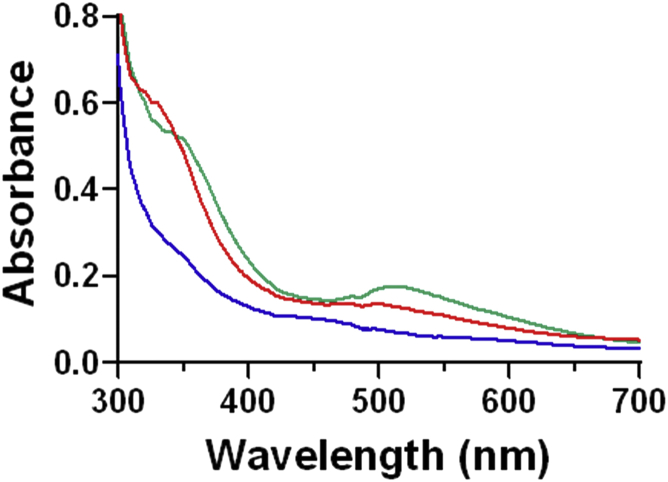
**Changes in the absorbance spectrum of the Mka HLP after addition of NO gas under anaerobic conditions.** The spectra were recorded before NO addition (*red*), after mixing with NO gas (*green*) and 1500 s after NO addition (*blue*). Samples contained 60 μM HLP. HLP, hemerythrin-like protein; Mka, *Mycobacterium kansasii*.

The Mka HLP was mixed with NO under anaerobic conditions. Analysis for reaction products in the mixture after this reaction indicated that the product was primarily NO_2_^−^ (Table 1). Intermediate species with distinct spectroscopic features were observed during this reaction. A species exhibiting a broad absorbance centered at 520 nm (Fig. 3) formed within the time of manual mixing of the Mka HLP with NO. The 520-nm absorbance feature decayed slowly over several minutes. Analysis for reaction products in the reaction mixture at this early time point, after formation of the 520-nm intermediate, revealed that most of the NO_2_^−^ had been formed, and that there was negligible additional formation during the decay of this intermediate. The total amount of NO_2_^−^ that was formed was approximately equivalent to the concentration of the Mka HLP. Thus, at best, stoichiometric conversion of NO was observed and the protein was unable to catalyze multiple turnovers. In these experiments, no reductant was added to the reaction mixture to prereduce the Mka HLP, and therefore, the results indicate that NO reacts with the diferric protein. When the reaction was repeated with the Mka HLP that was first reduced with dithionite to the mixed-valence state, NO_2_^−^ formation was significantly reduced (Table 1). This strongly suggests that the initial step in the anaerobic reaction is reductive nitrosylation of the diferric iron center by NO to a mixed-valence state. As such, when the protein was prereduced to the mixed-valence state by dithionite, this interfered with the initial reaction with NO.

**Table 1:** Reaction products of the Mka HLP with NO under anaerobic conditions

Sample	[Nitrite, NO_2_^−^], μM	[Nitrate, NO_3_^−^], μM
NO only	0	0
Mka HLP only	0	0
Mka HLP + NO	74 ± 7	8 ± 3
Dithionite-reduced Mka HLP +NO	18 ± 4	6 ± 1

Concentrations of nitrite and nitrate were determined by ion chromatography. The Mka HLP was present at 60 μM and NO at 450 μM in deoxygenated 50 mM MOPS, pH 7.5. Reactions were performed in triplicate.

**Figure 4:**
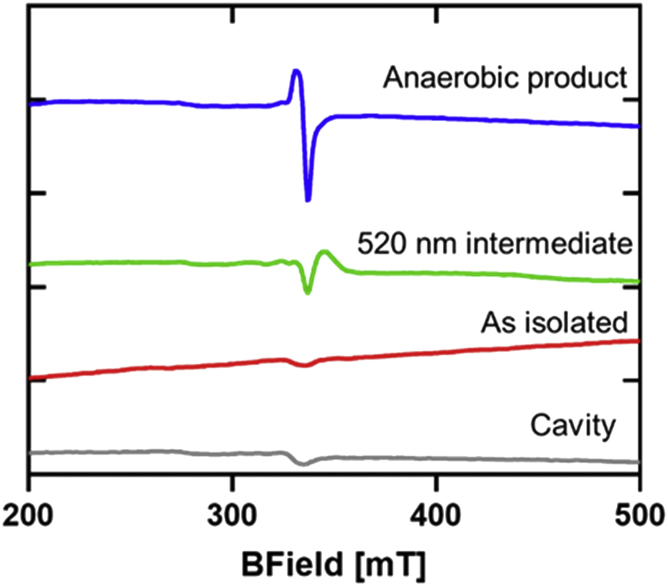
**EPR spectra of samples resulting from the anaerobic reaction of the Mka HLP with NO.** Samples contained 200 μM HLP. Reaction was initiated with addition of NO gas to form the 520-nm intermediate (*green*) and incubated at room temperature under anaerobic conditions for 30 min to form the anaerobic product (*blue*). The background signal from the cavity is *gray*. Spectra were collected at 15 to 17 K, at 10 G modulation amplitude, and 1 mW microwave power. HLP, hemerythrin-like protein; Mka, *Mycobacterium kansasii*.

**Figure 5:**
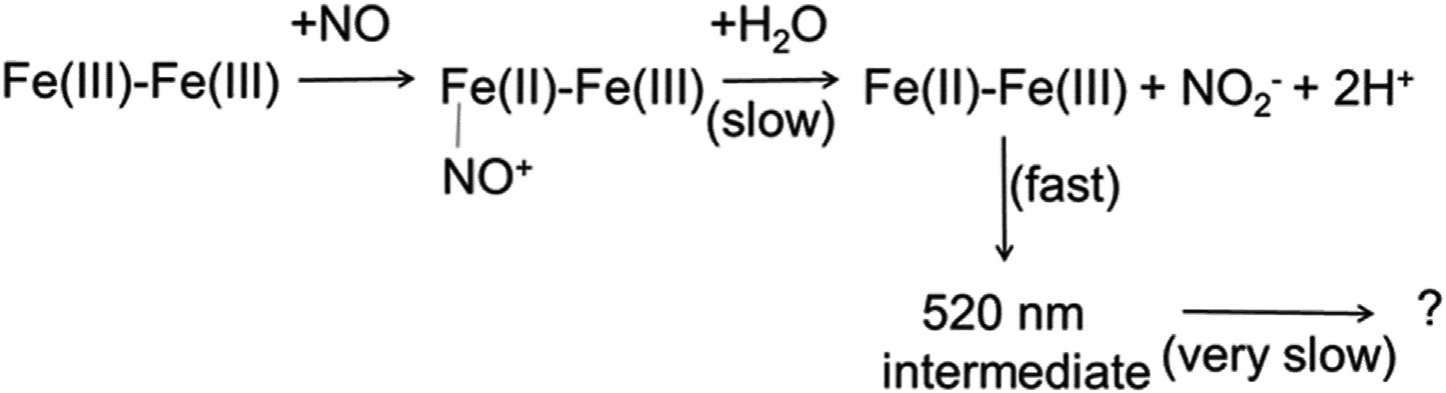
**Scheme of the anaerobic reaction of the Mka HLP with NO.** The bridging oxygen that interacts with the two irons is not shown for simplicity. HLP, hemerythrin-like protein; Mka, *Mycobacterium kansasii*.

The 520-nm intermediate in the anaerobic reaction was also trapped and analyzed by EPR spectroscopy. The spectrum indicated that the species is either a diamagnetic or an integer spin system. EPR analysis of the sample after decay of the 520-nm intermediate revealed a species with a signal that was easily power saturated at all temperatures (Fig. 4). This behavior suggests that the signal is an organic radical, perhaps an amino acid radical, and not related to the diiron product, which appears to be EPR silent and is possibly a diferric center. A possible mechanism for this conversion of NO to NO_2_^−^ that is consistent with these results is shown in Figure 5. A notable feature of this mechanism is that the reaction is initiated by reductive nitrosylation to form an Fe(II)-NO intermediate, which reacts with water to yield NO_2_^−^. While the latter reaction step has been observed for heme iron in proteins, including hemoglobin (15), this has not typically been seen in nonheme diiron proteins. For the Mka HLP, this allows the diiron site to oxidize NO.

### Aerobic reaction of Mka HLP with NO to form NO_3_^−^

**Figure 6:**
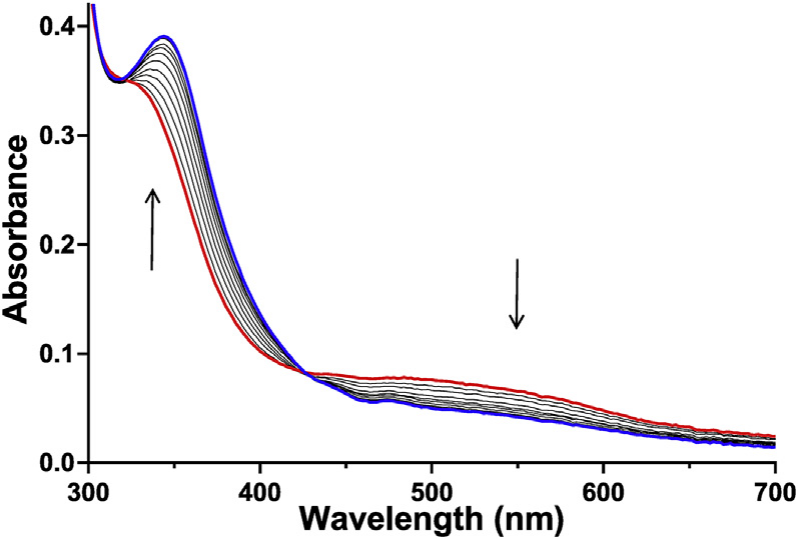
**Changes in the absorbance spectrum on aerobic addition of increasing amounts of NO.** The initial spectrum before NO additions is *red* and the final spectrum after NO additions is *blue*. The spectra resulting from incremental additions of NO are *black* and the direction of the spectral changes are indicated by *arrows*. NO, nitric oxide.

The primary change in the absorbance spectrum of the as-isolated Mka HLP on addition of NO under aerobic conditions is the formation of a weak absorbance feature in the 300 to 350 nm range that overlaps the shoulder of the 280 nm protein absorbance. There is also a broad absorption peak centered around 500 nm in the as-isolated protein, which decreases in intensity on reaction with NO. An aerobic titration of the Mka HLP with NO is shown in Figure 6.

Determination of the product of the reaction of the Mka HLP with NO under aerobic conditions required careful consideration. A significant experimental complication is that NO at high concentrations and under aerobic conditions reacts with O_2_ to form NO_2_^−^; a process known as NO autoxidation (20). Under these conditions, it is difficult to differentiate between NO_2_^−^produced by the Mka HLP and that produced from nonenzymatic autoxidation. However, the rate of NO autoxidation is second order with respect to NO concentration (20), and therefore, at micromolar NO concentrations, NO has a half-life of several minutes. For this reason, previous studies of enzymatic NO dioxygenase activity utilized low micromolar concentrations of NO to avoid the competing autoxidation (21, 22). Even so, it is not possible to completely eliminate nonenzymatic side reactions. To assure accurate interpretation of our results, controls and with all other reaction components except the Mka HLP were always performed to correct for background formation of NO_2_^−^.

When NO was mixed with the Mka HLP in air saturated buffer, analysis of the reaction mixture indicated that NO_3_^−^ was the primary product (Table 2). Some NO_2_^−^ was also present, but the amount of NO_2_^−^ that was formed was actually less than the background level in the absence of the protein, 290 μM *versus* 320 μM. An explanation for this is that in the presence of the Mka HLP, some of the NO was diverted from nonenzymatic NO autoxidation to be specifically converted to NO_3_^−^ by the Mka HLP. The maximum amount of NO_3_^−^ detected after completion of the reaction was at best stoichiometric with the concentration of Mka HLP. Thus, as observed for the anaerobic reaction, the Mka HLP cannot catalyze multiple turnovers of this aerobic reaction either.

**Table 2:** Reaction products of the Mka HLP with NO under aerobic conditions

Sample	[Nitrite, NO_2_^−^], μM	[Nitrate, NO_3_^−^], μM
NO only	320 ± 20	0
Mka HLP only	0	0
Mka HLP + NO	290 ± 7	59 ± 13

Concentrations of nitrite and nitrate were determined by ion chromatography. The Mka HLP was present at 60 μM and NO at 450 μM in air saturated 50 mM MOPS, pH 7.5. Reactions were performed in triplicate.

The mechanism of aerobic conversion of NO to NO_3_^−^ by the Mka HLP is unclear. In NO dioxygenase, which contains flavin and heme cofactors, the conversion of NO to NO_3_^−^ is initiated by O_2_ binding to a heme Fe(II) to generate an Fe(II)-O_2_ intermediate that then reacts with NO to ultimately form NO_3_^−^ *via* an O-bound ferric-peroxynitrite intermediate, Fe(III)-OONO. For this Mka HLP NO dioxygenase activity, we propose that the sequence of O_2_ and NO binding is reversed. For the Mka HLP, what can be said with certainty is that NO_3_^−^ formation is produced in the presence of O_2_, whereas NO_2_^−^ is the sole product of the anaerobic reaction. In each of these cases, the mechanisms of transformation of NO to NO_3_^−^ and NO to NO_2_^−^ by the oxo-bridged diiron center are highly unusual, if not novel.

### NO peroxidase activity of the Mka HLP

Two observations suggested the possibility that the Mka HLP could have NO peroxidase activity. First, it was previously shown that Mka HLP possessed weak catalase activity (4). This indicated that H_2_O_2_ could bind to at least one of the irons in the diiron site. Second, the anaerobic reaction with NO, described in this study, indicated that NO can reduce one of the irons by reductive nitrosylation. This suggests the possibility that NO could serve as the electron donating substrate in a peroxidase reaction. To test for this activity, the Mka HLP was mixed with H_2_O_2_ and NO. The reaction was performed under anaerobic conditions to minimize NO autoxidation. These conditions also approximate physiological conditions for a *Mycobacterium* inside the macrophage or granuloma during infection. The O_2_ levels within the *Mycobacterium* can be extremely low in this hypoxic environment where high levels of NO and H_2_O_2_ are generated to attack the bacterium (17, 23, 24). Analysis of the reaction mixture after completion of the reaction yielded NO_2_^−^ as the primary product, with product formation well in excess of the concentration of the Mka HLP (Table 3). Thus, in contrast to the other single-turnover activities described above for the anaerobic NO oxidase and aerobic NO dioxygenase activities, the NO peroxidase reaction is an actual multiturnover enzymatic activity of the Mka HLP.

**Table 3:** Catalytic Mka HLP-dependent nitrite production *via* a peroxidase reaction

Conditions	Sample	[NO_2_^−^] (μM)	HLP-dependent [NO_2_^−^] (μM)	Enzymatic turnovers
Anaerobic	H_2_O_2_ + NO	51 ± 1		
	HLP + H_2_O_2_ + NO	95 ± 3	44	8.8
Aerobic	H_2_O_2_ + NO	129 ± 2		
	HLP + H_2_O_2_ + NO	143 ± 3	14	2.8

Reactions were performed in 50 mM phosphate, pH 7.5, at 25 °C in room air or an anaerobic glove box. The samples with HLP were 5 μM in diiron site concentration. H_2_O_2_ was present at 1 mM and the reaction was initiated by addition of 200 μM NO. The reaction time was 10 min. Reactions were performed in triplicate. Methods for quenching the reaction, removal of excess H_2_O_2_ and nitrite determination are described under Experimental procedures.

This reaction was repeated under aerobic conditions. In these aerobic reactions, NO_2_^−^ production by NO autoxidation was observed, as expected; nevertheless, it was still possible to observe Mka HLP-dependent, H_2_O_2_-dependent NO_2_^−^ production (Table 3). While the amount is less than was observed anaerobically, it still represents multiple enzymatic turnovers and indicates that the Mka HLP-dependent peroxidase activity can compete with the spontaneous NO autoxidation reaction under aerobic conditions.

**Figure 7:**
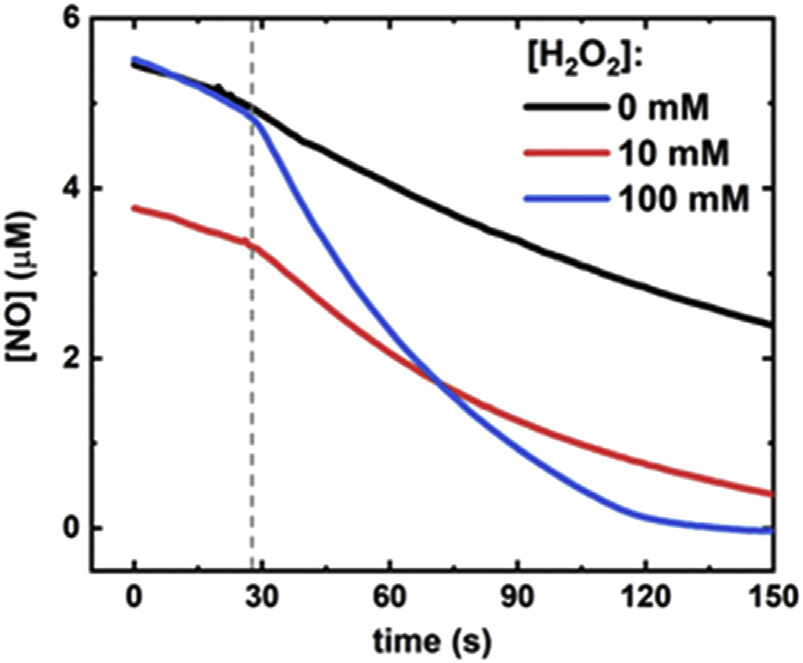
**Mka HLP and H**_**2**_**O**_**2**_**-dependent NO consumption.** Representative traces of NO consumption as monitored by an NO electrode. Samples were in degassed 50 mM phosphate, pH 7.5 at room temperature and contained the concentration of H_2_O_2_ indicated in the legend. The *dashed gray line* indicates when 1 μM HLP was added to each sample. HLP, hemerythrin-like protein; Mka, *Mycobacterium kansasii*; NO, nitric oxide.

To further characterize this NO peroxidase activity, NO consumption by the Mka HLP under anaerobic conditions was monitored using an NO electrode (Fig. 7). A background rate of NO consumption was observed prior to addition of the Mka HLP that is attributed to nonenzymatic reaction of NO with H_2_O_2_ or residual O_2_ in the chamber. Addition of the Mka HLP resulted in an immediate increase in the NO consumption rate. This Mka HLP-dependent rate increased with increasing H_2_O_2_ concentration (Table 4). In addition, 1 μM HLP reproducibly consumed 5 to 6 μM NO within minutes under these conditions. This result provides further evidence that NO consumption is catalytic with multiple turnovers with respect to NO consumption.

**Table 4:** Initial rates of NO consumption in the absence (-HLP) and presence (+HLP) of the Mka HLP, and varying concentrations of H_2_O_2_

[H_2_O_2_]	Initial rates of NO consumption (nM NO/s)		
	−HLP	+HLP	HLP-dependent
0 mM H_2_O_2_	17 ± 2	30 ± 1	13
10 mM H_2_O_2_	15 ± 1	36 ± 6	21
100 mM H_2_O_2_	25 ± 5	94 ± 10	69

Reactions were performed in deoxygenated 50 mM phosphate, pH 7.5, at room temperature. The samples contained 5 to 6 μM NO and H_2_O_2_ as indicated. The Mka HLP-dependent reaction was determined by subtracting the background NO consumption rate with that observed within 5 s of adding 1 μM Mka HLP. Reactions were performed in triplicate.

The Mka HLP does exhibit some weak catalase activity to produce O_2_ (4). Thus, it is possible that even under anaerobic conditions, nonenzymatic autoxidation of NO to form NO_2_^−^ could result from the reaction of the catalase-produced O_2_. Therefore, it was important to rule this out as a possible alternative explanation to actual peroxidase activity. Comparison of the NO consumption rate in Table 4 with that of the catalase activity of the Mka HLP at 10 mM H_2_O_2_ precludes this possibility. Addition of the Mka HLP increased the NO consumption rate under these conditions from 15 ± 1 nM NO/s to 36 ± 6 nM NO/s. The Mka HLP-dependent NO consumption rate is therefore 21 nM NO/s. By comparison, the steady-state catalase activity of 1 μM Mka HLP under these conditions was 4.1 ± 0.3 μM H_2_O_2_/s, which is equivalent to production of 2 μM O_2_/s. Because the initial NO consumption rates were calculated within 5 s of HLP addition, only up to 10 μM O_2_ could accumulate within this interval. The rate of NO autoxidation at 25 °C was calculated using Equation 1 (25). The fastest NO consumption rate at 10 μM O_2_ and 5 μM NO was calculated as 2.3 nM NO/s or approximately tenfold slower than the observed rate of 21 nM NO/s in the presence of the Mka HLP. This analysis confirms the conclusion that the observed NO consumption and subsequent nitrite formation is the result of an enzymatic NO peroxidase activity and not an artifact related to the catalase activity of the Mka HLP.

(1) $$-\text{d}\left[\text{NO}\right]/\text{dt}=9\times 10^{6}{\text{M}}^{-2}{\text{s}}^{-1}{\left[\text{NO}\right]}^{2}\left[{\text{O}}_{2}\right]$$

### Steady-state kinetic analysis of Mka HLP-dependent NO peroxidase activity

**Figure 8:**
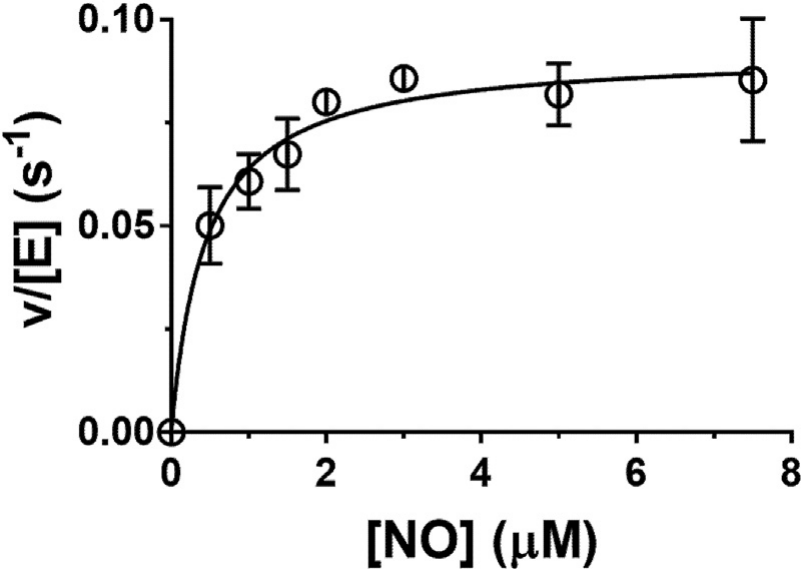
**Steady-state kinetic analysis of NO consumption during the NO peroxidase reaction catalyzed by the Mka HLP.** The concentration of NO was monitored using an NO electrode. NO was varied in the presence of 100 nM Mka HLP and 100 mM H_2_O_2_ in 50 mM potassium phosphate, pH 7.5, at 20 °C, under anaerobic conditions. Points are the average of three replicates. The line is a fit of the data to Equation 2 with an R^2^ of 0.98. HLP, hemerythrin-like protein; Mka, *Mycobacterium kansasii*; NO, nitric oxide.

Kinetic studies of the NO peroxidase reaction could monitor formation of either the nitrite product or consumption of the NO substrate. There is not a continuous assay available with which to monitor NO_2_^−^ production; however, the rate of consumption of the NO substrate could be monitored using an NO electrode as in Figure 7. The reactions were initiated by addition of the Mka HLP to the mixture containing NO and H_2_O_2_. The order of addition of H_2_O_2_ and HLP to NO did not affect the rate of the reaction. Initial rates of NO consumption, corrected for background nonenzymatic loss of NO, were determined at different concentrations of NO to determine the *K*_m_ for NO and *k*_cat_ for the reaction (Fig. 8). The results indicate that the enzyme is saturated at low micromolar concentrations. Analysis of the data yielded values *k*_cat_ of 9.2 ± 0.3 × 10^−2^ s^−1^ (5.5 min^−1^), *K*_M_ of 0.44 ± 0.08 μM, and a *k*_cat_/*K*_M_ of 2.3 × 10^5^ M^−1^s^−1^.

The studies described in Table 4 confirmed that nitrite was the product of the reaction. While it would be desirable, it was not possible to also monitor NO_2_^−^ production in parallel with NO consumption. As described under Experimental procedures, for each time point the reaction would need to be quenched and then analyzed by the multistep chemical reactions of the Griess assay. The limit of detection of nitrite by the Griess assay is approximately 2.5 μM nitrite. This is far greater than the *K*_m_ for NO of 0.44 μM. Furthermore, H_2_O_2_ interferes with the Griess reaction further decreasing its sensitivity. As such, it would not be possible to monitor the steady-state reaction by monitoring nitrite production in the relevant range of substrate concentration. One cannot simple remove H_2_O_2_ by addition of catalase as that would produce O_2_, which would then react nonenzymatically with NO to produce nitrite. Similarly, preparations of samples for analysis by ion chromatography also require H_2_O_2_ removal, an anaerobic overnight incubation, and chromatography for each sample. This is why the nitrite product results presented in Table 1, Table 2, Table 3, Table 4, which were performed at higher concentrations of NO, were end point determinations after the completion of the reaction, which were corrected for nonenzymatic background reactions.

### Possible NO peroxidase mechanisms

**Figure 9:**
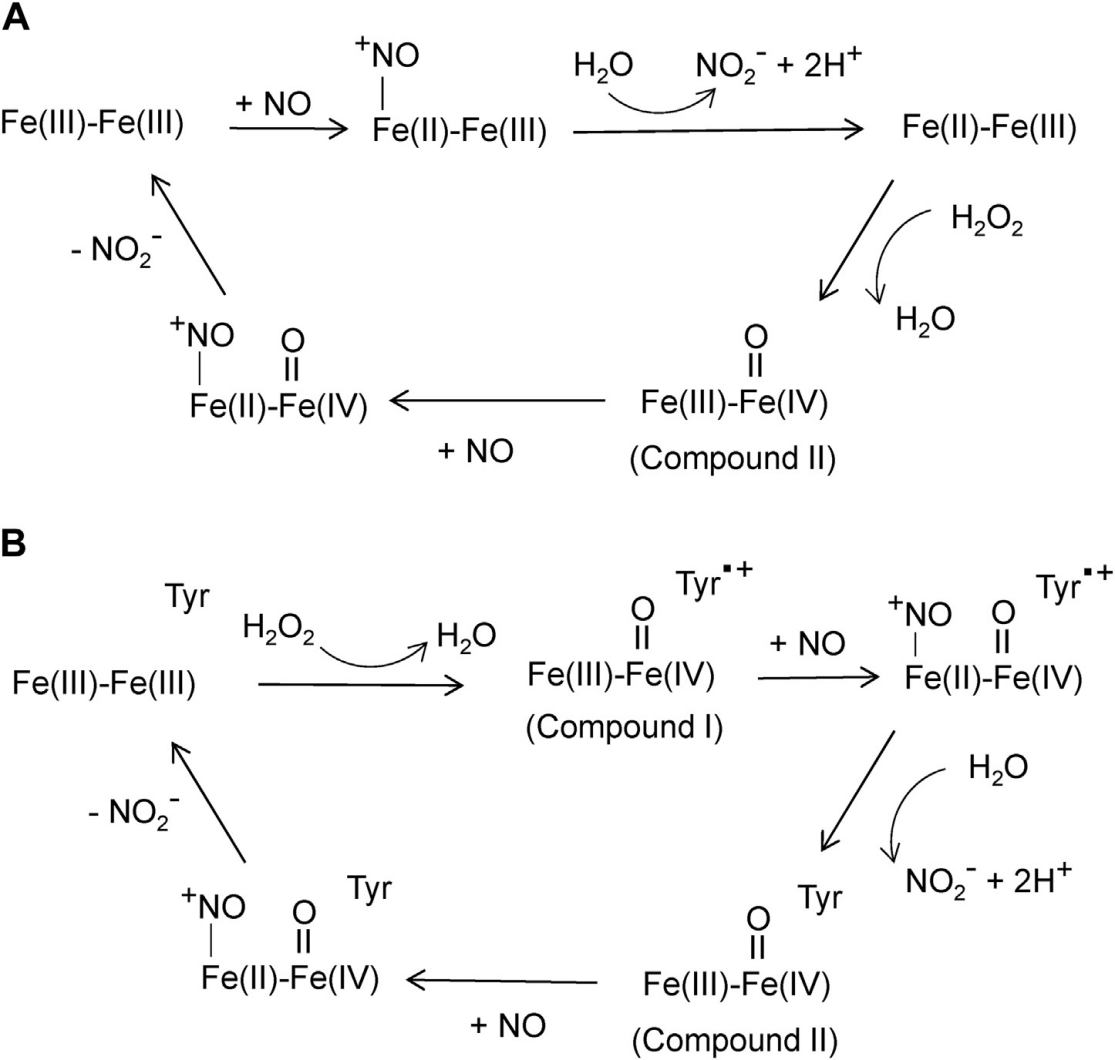
**Proposed mechanisms for the NO peroxidase reaction that is catalyzed by the Mka HLP.** Mechanism A utilizes only the oxo-bridged diiron site. Mechanism B also utilizes the Tyr residue that provides a ligand for iron. The bridging O is not shown for simplicity. HLP, hemerythrin-like protein; Mka, *Mycobacterium kansasii*; NO, nitric oxide.

Two possible mechanisms for the NO peroxidase reaction that is catalyzed by the Mka HLP are proposed (Fig. 9). Each is consistent with the sum of the data and observations from this study. In each, the starting and end points of the reaction cycle are the Fe(III)-Fe(III) state. The bridging O is not shown for simplicity. One mechanism utilizes only the diiron site for catalysis (Fig. 9*A*). The initial two steps are the same as proposed for the anaerobic reaction with NO (see Fig. 5). The reaction is initiated by reductive nitrosylation to yield NO_2_^−^. In contrast to the anaerobic mechanism in Figure 5, H_2_O_2_ then reacts with the mixed-valence species to generate a Compound II-like species plus water. This is followed by a second NO reacting with the Fe(III) of Compound II to form a second NO_2_^−^ and regenerate the diferric site. The other possible mechanism is patterned after the mechanism for heme-dependent peroxidases (Fig. 9*B*). The first step in this mechanism is reaction with H_2_O_2_ to yield a Compound I-like intermediate. Compound I in heme iron sites is typically described as a ferryl Fe(IV)=O with a porphyrin cation radical. In this proposed mechanism for the Mka HLP with nonheme irons, the cation radical is centered on the Tyr that provides a ligand for one of the irons. This intermediate then undergoes reductive nitrosylation by NO, which leads to formation of a NO_2_^−^ and oxidation of the ferrous iron by the Tyr radical. This yields a Compound II-like intermediate that reacts with a second NO to generate a second NO_2_^−^, as also occurred in the other proposed mechanism.

## Discussion

The Mka HLP is a representative of a distinct class of HLPs found in infectious mycobacteria. That distinction was originally based on the sequence of the proteins. Hemerythrins function as oxygen carriers or storage proteins. The other HLPs have a variety of other functions. These include activity as sensors for oxygen (26) or iron (27), signal transduction (28) and repair of Fe-S centers (29). Characterization of the Mka HLP as an NO peroxidase further distinguishes it based on function. While each of these proteins possess an oxo-bridged diiron site, the identity and pattern of amino acid residues that provide ligands for the irons in the mycobacterial HLPs are distinct from the others. This is likely the basis for the unprecedented reactivity of the Mka HLP. The overall structure of the Mka HLP also differs from that of hemerythrin and other HLPs. It is a monomer with a core structure of five α-helices, rather than four α-helices. The majority of hemerythrins are multimers of six or eight four-helix subunits (2, 30). In the other HLPs, the four-helix domain is fused to another protein domain that dictates its function. The Mka HLP is a monomer, which is not fused to another protein domain.

The characterization of the NO peroxidase activity of the Mka HLP is not only an unprecedented activity for a hemerythrin or HLP, but also unexpected as peroxidases typically use heme cofactors to catalyze their reactions. Furthermore, the Mka HLP is a high-affinity NO peroxidase with a submicromolar *K*_m_ for NO. Similar micromolar or submicromolar *K*_M_ values have been reported for other NO detoxifying enzymes, including NO dioxygenases (10, 21), and NO reductases (31, 32, 33). This high affinity for NO is consistent with a role of the Mka HLP to scavenge NO at low NO concentrations to prevent accumulation of this potential toxic compound. The *K*_m_ value determined for NO in this study is in the range of concentration of NO needed for nitrosative stress (34). It is also consistent with the upregulation of this enzyme after phagocytosis by macrophages, which will expose the bacterium to NO. The high affinity could also allow this reaction to persist, even under aerobic conditions, as the Mka HLP-catalyzed peroxidase reaction was shown to be able to compete with the spontaneous NO decay under aerobic conditions.

In addition to the ability to catalyze an NO peroxidase reaction, the oxo-bridged diiron site of the Mka HLP exhibits chemical reactivity that is not typically seen in hemerythrins and other HLPs. The two irons are not readily reduced to a diferrous form, but instead to a stable mixed-valence species. Initial reaction with NO results in reductive nitrosylation, a process before only seen with heme iron. Furthermore, the mixed-valence state could not be readily reduced to the diferrous state that is commonly seen as an intermediate form of hemerythrins and other HLPs. These unusual properties of the Mka HLP are likely related to the nature of the amino acid side chains that coordinate the two irons and their geometry. This combination of amino acid residue ligands is unique to mycobacterial HLPs (1, 7). This information points to a novel mechanism for the catalysis of the peroxidase reaction by a diiron center. Taking into account the novel reactivity described above, two possible mechanisms for the reaction are proposed (Fig. 9) that are each consistent with the data presented herein.

The biological relevance of the Mka HLP goes beyond removal of NO. The NO peroxidase activity of the Mka HLP is well suited for a mycobacterial host. The two substrates for the reaction, H_2_O_2_ and NO, are each produced in macrophages to defend against an infectious *Mycobacterium* (35, 36). This occurs in a relatively hypoxic environment similar to the anaerobic conditions under which the Mka HLP was studied and shown to catalyze the peroxidase reaction. The NO peroxidase activity converts H_2_O_2_ to water and NO to nitrite, thus neutralizing both of the molecules used as the host defense mechanism. Furthermore, production of nitrite can also be advantageous, as nitrite is a signaling molecule in mycobacteria that slows the growth of the organism under hypoxia conditions (17, 23, 24). Thus, the action of the Mka HLP not only protects the host bacterium from oxidative and nitrosative damage, but also allows the host to adapt to the hostile hypoxic environment by virtue of the nitrite produced by the peroxidase reaction.

These studies provide a framework for future structure–function studies to determine the precise contributions of each of the amino acid ligands, as well as other residues in the protein that are unique to mycobacterial HLPs, to the unusual reactivity describe herein. The results also provide a rationale for physiologic studies of *M. kansasii*, as well as other infectious mycobacteria. For example, it is known that the gene for this protein is upregulated in *M. tuberculosis* after phagocytosis of the bacterium by macrophages. This raises the question of what might happen if the gene were knocked out or inactivated. Once the consequences of this are understood, it could further implicate these mycobacterial HLPs as potential targets for drugs to combat tuberculosis and other mycobacterial infections.

## Experimental procedures

### Protein expression and purification

The methods for expression and purification of the Mka HLP were as described previously (4). One difference is that the previous study used the gene that had been cloned from *M. kansasii*. In the current study, the protein was expressed using a commercially synthesized gene (Genewiz) that was codon-optimized for expression in *Escherichia coli* and to which an N-terminal His tag was added. This was cloned into a pET15b vector and transformed into *E. coli* Rosetta2(DE3) cells.

### Preparation of solutions

Dithionite (Na_2_S_2_O_4_) solutions were prepared by dissolving Na_2_S_2_O_4_ powder with deoxygenated water or buffer in an anaerobic glovebox. Dithionite solutions were quantified by UV-visible absorption spectroscopy using a known extinction coefficient (ε_315_ = 8 mM^−1^ cm^−1^).

Solutions of NO were prepared by two alternative methods. Saturated solutions of NO were prepared by bubbling NO gas into solution after passage through a NaOH solution to remove the impurities. Alternatively, the NO precursor PROLI NONOate (Cayman Chemical) was used. It was prepared by dissolving PROLI NONOate into 0.01 N NaOH solution and quantitated from the absorbance at 252 nm (ε = 8400 M^−1^ cm^−1^). Each molecule of the NONOate released two NO molecules after addition to the buffer.

### Sample preparation

Aerobic samples were generated using air-saturated 50 mM MOPS or 50 mM phosphate at pH 7.5. Anaerobic samples were generated in a Genesis glovebox (Vacuum Atmospheres Company) in an anaerobic N_2_ atmosphere. Buffers containing 50 mM MOPS or 50 mM phosphate at pH 7.5 were deoxygenated by three vacuum and N_2_ purge cycles on a Schlenk line. Reactions were initiated by addition of NO gas in the form of buffered NO. Buffered NO in septum-sealed headspace vials was transferred through the septum and to the samples by use of a 100-μL Hamilton syringe. Samples containing NO gas were prepared in a 2-mL septum sealed Starna cuvette. Reactions with NO gas were initiated by replacing the headspace of the cuvette with purified NO gas and inverting the cuvette to introduce NO into solution. The solution NO concentration in samples prepared in this manner was 1200 μM NO. UV-vis absorption spectra were collected on an Ocean Optics USB 2000+ spectrometer in the glovebox to prevent contamination with O_2_.

### Determination of nitrite and nitrate concentrations formed by reaction with NO.

Determination of nitrite concentrations during assays was achieved using the Griess assay (37). For determination of the time course for nitrite formation, samples were collected by quenching 100 μl aliquots of each reaction sample with 50 μl of deoxygenated Griess reagent R1 (1% sulfanilamide in 5% H_3_PO_4_) and mixing by pipetting. Development of the Griess assay was then immediately initiated thereafter by addition of 50 μl of deoxygenated Griess reagent R2 (0.1% napthylethylenediamine dihydrochloride in water). The concentration was determined by using an extinction coefficient, ε_542_ = 50 mM^−1^ cm^−1^ as well as from a standard curve that was generated from reactions with known concentrations of nitrite.

Quantitation of nitrite in samples containing H_2_O_2_ during the peroxidase reaction required additional preparation steps because H_2_O_2_ concentrations greater than 1 mM interfere with the Griess assay. After incubation of these samples in the glovebox, the samples were purged with N_2_ gas to remove excess NO. Afterward, excess H_2_O_2_ was removed by addition of 50 ug of catalase and incubated at room temperature for 5 min. The H_2_O_2_ concentration was determined spectrophotometrically by monitoring the absorbance at 240 nm using the extinction coefficient of *ε*_240_ = 43.6 M^−1^ cm^−1^. The resulting samples were then analyzed by Griess assay as described above.

Formation of nitrite and nitrate was also quantitated by ion chromatography. Samples for ion chromatography were prepared and incubated overnight in an anaerobic glovebox. This incubation ensured complete removal of solution NO from the samples. After overnight incubation, the samples were removed from the glovebox and the protein that was present was removed using three MWCO Amicon microcentrifugal filters (Millipore). The filtrate was then analyzed by ion chromatography for nitrite and nitrate using a Dionex Integrion High-Pressure Ion Chromatography (ThermoScientific) equipped with a 4 mm anionic exchange column (IonPac AS20), suppressor (Dionex ADRS 600 Suppressor) and a conductivity detector, operated at constant voltage (4.0 V). The sample loop was 20 μl and was first degassed in an internal oven at 30 °C and then carried through the column by 35 mM NaOH (ultrapure, carbonate free, Acros Organics). The elution times under the conditions studied were 4 min and 4.9 min for nitrites and nitrates, respectively.

### Electron paramagnetic spectroscopy

X-band (9.51 GHz) EPR spectra were acquired using a Freiberg Instrument Miniscope MS5000 spectrometer equipped with an Advanced Research Systems LTR helium flow cryostat. Standard collection parameters were 1.0 mT modulation amplitude and a 100 s sweep time. Temperature and microwave powers during collection are noted in the figure captions.

### NO electrode studies

Experiments that monitored NO consumption were performed in a multiport measurement chamber with a magnetic stir bar (World Precision Instruments). The reactions were performed in 1.5 ml of 50 mM phosphate buffer, pH 7.5. The buffer was degassed prior to the experiment by bubbling N_2_ gas through a 22 G needle threaded through the cap of the reaction chamber and into the buffer for 5 to 10 min. The needle was removed prior to addition of NO to the chamber and the cap lowered to the liquid level to eliminate the headspace in the reaction vessel. The fastest rotation rate on the magnetic stir plate resulted in the smallest background NO consumption rate. Under this configuration, the NO electrode was calibrated using quantified PROLI-NONOate solutions. Final HLP and H_2_O_2_ concentrations in each NO electrode experiment are listed in the figure captions.

### Steady-state kinetic studies

An NO electrode was used to monitor [NO] as described above during the time course of the NO peroxidase reaction catalyzed by the Mka HLP. For steady-state kinetic analysis of the reactions, the initial linear rate of the decrease in [NO] was determined and plotted. Data were fit to Equation 2 where v is the initial rate, S is NO, and E is the Mka HLP.

(2) $$\text{v}/\left[\text{E}\right]=k_{\text{cat}}\left[\text{S}\right]/\left(K_{\text{m}}+\left[\text{S}\right]\right)$$

## Data availability

All data are contained within the manuscript.

## Conflict of interest

The authors declare that they have no conflicts of interest with the contents of this article.
